# Role of oxidative stress in the physiology of sensitive and resistant *Amaranthus palmeri* populations treated with herbicides inhibiting acetolactate synthase

**DOI:** 10.3389/fpls.2022.1040456

**Published:** 2023-01-06

**Authors:** Mikel Vicente Eceiza, María Barco-Antoñanzas, Miriam Gil-Monreal, Michiel Huybrechts, Ana Zabalza, Ann Cuypers, Mercedes Royuela

**Affiliations:** ^1^ Institute for Multidisciplinary Research in Applied Biology (IMAB), Public University of Navarre, Pamplona, Spain; ^2^ Environmental Biology, Centre for Environmental Sciences, Hasselt University, Diepenbeek, Belgium

**Keywords:** oxidative stress, nicosulfuron, herbicide mode of action, acetolactate synthase, *Amaranthus palmeri*

## Abstract

The aim of the present study was to elucidate the role of oxidative stress in the mode of action of acetolactate synthase (ALS) inhibiting herbicides. Two populations of *Amaranthus palmeri* S. Watson from Spain (sensitive and resistant to nicosulfuron, due to mutated ALS) were grown hydroponically and treated with different rates of the ALS inhibitor nicosulfuron (one time and three times the field recommended rate). Seven days later, various oxidative stress markers were measured in the leaves: H_2_O_2_, MDA, ascorbate and glutathione contents, antioxidant enzyme activities and gene expression levels. Under control conditions, most of the analysed parameters were very similar between sensitive and resistant plants, meaning that resistance is not accompanied by a different basal oxidative metabolism. Nicosulfuron-treated sensitive plants died after a few weeks, while the resistant ones survived, independently of the rate. Seven days after herbicide application, the sensitive plants that had received the highest nicosulfuron rate showed an increase in H_2_O_2_ content, lipid peroxidation and antioxidant enzymatic activities, while resistant plants did not show these responses, meaning that oxidative stress is linked to ALS inhibition. A supralethal nicosulfuron rate was needed to induce a significant oxidative stress response in the sensitive population, providing evidence that the lethality elicited by ALS inhibitors is not entirely dependent on oxidative stress.

## 1 Introduction

Weed control is and has always been a major challenge for agriculture worldwide. Among all the implemented methods to minimise or avoid the many problems derived from weeds, herbicides continue being the most widely used tools for weed control (Edwards and Hannah, 2014). Herbicides inhibit specific molecular target sites within plant biological pathways and processes, and are classified into groups according to their target. Acetolactate synthase (ALS) inhibiting herbicides are some of the most popular postemergence herbicides worldwide owing to their efficacy and relatively low environmental impact (Mazur and Falco, 1989). ALS inhibitors form a diverse herbicide group with several families, where sulfonylureas are included. The inhibition of the key enzyme ALS in the pathway of biosynthesis of the branched-chain amino acids, isoleucine, leucine, and valine, is the common mechanism of action of all these herbicides (Zhou et al., 2007). Despite the extended usage of ALS inhibitors, and the fact that this mechanism of action was discovered decades ago, their mode of action has not been completely elucidated, even though some physiological effects are known. Among other effects, ALS inhibition leads to growth arrest, impairment of carbohydrate utilisation by roots and carbohydrate accumulation in leaves (Zabalza et al., 2008; Orcaray et al., 2011), alterations in phosphorus metabolism (Dragicević et al., 2011), and decrease of protein levels in favour of free amino acid pool (Shaner and Reider, 1986; Scarponi et al., 1995; Scarponi et al., 1997; Sidari et al., 1998; Zulet et al., 2013). Another reported effect, albeit less studied, is oxidative stress.

Oxidative stress develops as a result of an imbalance between reactive oxygen species (ROS) and antioxidant mechanisms in favour of the former, which manifests in oxidative damage in biomolecules and/or a stimulation of antioxidant systems (Demidchik, 2015). This imbalance is developed in response to almost all biotic and abiotic stresses. Under adequate conditions, ROS production and antioxidant activity are balanced, but stressful conditions usually lead to oxidative stress. As happens with other herbicides that inhibit amino acid biosynthesis, such as glyphosate (Ahsan et al., 2008; Gomes and Juneau, 2016; Ostera et al., 2016; de Freitas-Silva et al., 2017; Eceiza et al., 2022) or glufosinate (Takano et al., 2019), ALS inhibitors are thought to cause moderate oxidative stress secondarily, not being the main cause of plant death (Zabalza et al., 2007; Caverzan et al., 2019).

Over-reliance on herbicides can lead to the selection for resistant weeds (Yu and Powles, 2014) and, as a consequence of the massive usage of ALS inhibitors, 170 species have evolved populations with resistance to ALS-inhibiting herbicides (Heap, 2022), which makes them the group of herbicides with the highest number of weed species containing at least one resistant population (Heap, 2022). One of those species is *Amaranthus palmeri* S. Watson, a C4 weed whose fast growth, seed dispersal and extremely high genetic variability, makes it prone to the development of resistance to herbicides, make its control really difficult. Resistance mechanisms can be classified in target-site resistances (TSR) and non-target-site resistances (NTSR), depending on whether resistance is based in the target molecule or is a generalist mechanism developed independently of the target. In *A. palmeri*, although NTSR to ALS inhibitors mechanisms have been reported (Nakka et al., 2017), resistance to ALS inhibitors is more commonly TSR; specifically, substitution mutations in the *ALS* gene that make the enzyme insensitive to the action of ALS inhibitors. This is also the main mechanism detected in populations of other species like *Digitaria sanguinalis* (Murphy and Tranel, 2019) or *Papaver rhoeas* (Rey-Caballero et al., 2017).

*Amaranthus palmeri* is native to America, where the majority of resistant populations have been found: Kansas (USA) (Sprague et al., 1997; Chaudhari et al., 2020), Argentina (Larran et al., 2017), Brazil (Küpper et al., 2017), etc. This species has been introduced in other continents and, since 2007, it is present in Spain (Torra et al., 2020). Moreover, resistant biotypes to ALS inhibitors have been recently found in Catalonia, Spain, due to several amino acid substitutions in the *ALS* gene in positions Pro-197, Trp-574, and Ser-653 (Torra et al., 2020). This resistance mechanism is well characterised, but the possible physiological implications of this mutation and the physiological effects triggered by the exposure to an ALS inhibiting herbicide in these resistant weeds is not known. The genetic similarity of these *A. palmeri* populations with TSR and the sensitive population of reference (only ALS point mutations), gives the opportunity to study the physiological responses of resistant biotypes to ALS inhibitors, and specifically the role of oxidative stress in the mode of action of ALS inhibitors, and in the resistance to these herbicides.

As stated before, ALS inhibitors have been previously linked to oxidative stress. However, three key aspects of this connection remain to be elucidated. The first one is the importance of oxidative stress in the toxicity of these herbicides and the role that this stress may play in their mode of action. The second one refers to the origin of the oxidative stress triggered by herbicide application. How oxidative stress is generated, and whether it is caused in some way by ALS inhibition or if it is an independent secondary effect of the herbicide target, is not well known. Finally, the third refers to the resistant weed populations. Basal oxidative status of resistant individuals may be different from that of sensitive individuals. Whether or not it is different, sensitive and resistant plants may also respond differently to herbicide treatment in terms of oxidative stress. In this sense, comparing the oxidative stress produced by an ALS inhibitor in a sensitive population and in a resistant population by a TSR mechanism, where the target enzyme is insensitive to the herbicide, would allow evaluating if ALS inhibition is somehow the cause of oxidative stress. In addition, the oxidative status of these populations can also be analysed and contrasted with the response to the herbicide.

In this study, the main objective was to evaluate the impact of *ALS* gene mutations and of ALS inhibitor treatment on oxidative stress. To this aim, the basal pattern and the response of a sensitive and a resistant population of *A. palmeri* to different rates of the ALS inhibitor nicosulfuron, a sulfonylurea, were evaluated, in terms of oxidative stress and antioxidant responses. This study will help to establish the possible physiological alterations of the oxidative status in sensitive and resistant plants, the physiological importance of this oxidative stress and whether ALS inhibition is causing the oxidative stress.

## 2 Materials and methods

### 2.1 Plant material and treatment application

The seeds of the *A. palmeri* populations nicosulfuron-sensitive (S) and resistant (R) were kindly provided by Dr. Joel Torra (University of Lleida, Catalonia, Spain) and were originally collected from Lleida (Spain). In R plants, resistance is supposed to be conferred by a mutation in Ser-653 and/or Trp-574 (Torra et al., 2020).

Plants were cultivated and grown hydroponically. Briefly, seeds were surface sterilized prior to germination (Labhilili et al., 1995). For germination, seeds were incubated for 4 days at 4 °C in darkness. Then, they were maintained for 60 h in a light/darkness cycle of 16 h/8 h at a temperature of 30 °C/18 °C. After germination, plants were transferred to aerated 2.7 L hydroponic tanks in a phytotron (day/night, 16 h/8 h; light intensity, 0.5 mmol s^-1^ m^-2^ PAR; temperature, 22/18 °C; relative humidity, 60/70%). The plants remained in the vegetative phenological stage throughout the entire course of the experiment. The full-strength Hoagland solution with 15 mM KNO_3_ (Hoagland and Arnon, 1950) was used as nutrient solution. All the analytical determinations were made on plants in the vegetative phenological stage.

Plants were treated after reaching the growth stage defined as BBCH 14.35 (19-22 days old). Nicosulfuron (commercial product: Samson^®^, Key, Tárrega, CAT, Spain) was applied at two different rates: recommended field rate (FR) or 3 times FR (3FR), being FR 0.06 kg ha^-1^ (Huang et al., 2019). The herbicide was sprayed using an aerograph (model Definik, Sagola, Vitoria-Gasteiz, EUS, Spain) connected to a compressor (model Werther one, Breverrato, Italy; 60 W, 10 L m^-1^, 2.5 bar). On each population, untreated plants (rate 0, control) were sprayed with water using the same aerograph and compressor.

### 2.2 Analytical determinations

One week after herbicide treatment, leaves were sampled, frozen in liquid N_2_ and stored at -80 °C. Leaf samples were powdered with a Retsch mixer mill (MM200, Retsch, Haan, Germany) and the amount of tissue needed for each analytical determination was separated. Several treated and untreated plants were left in the phytotron to check the lethality of the applied nicosulfuron rates.

#### 2.2.1 DNA analysis: Derived Cleaved Amplified Polymorphic Sequences (dCAPS) assay for the resistant mutation

Genomic DNA was extracted from previously ground *A. palmeri* leaves (0.1 g), as described before (Fernández-Escalada et al., 2016). DNA was quantified and analysed using a Synergy™ HT Multi-Detection Microplate Reader (BioTek Instruments Inc., Winooski, VT, USA) and its quality was checked using 1% agarose gel electrophoresis. Extracted gDNA was used to perform the Derived Cleaved Amplified Polymorphic Sequences (dCAPS) assay for resistant mutations in two *ALS* positions: Trp-574 and Ser-653, to confirm which mutation(s) occurred.

The dCAPS analyses were conducted to determine the possible mutant ALS alleles, as described before (Barco-Antoñanzas et al., 2022).

#### 2.2.2 H_2_O_2_ detection

Hydrogen peroxide (H_2_O_2_) concentration was determined using the Amplex™ Red Hydrogen Peroxide/Peroxidase Assay Kit (Invitrogen, Thermo Fisher Scientific, Carlsbad, CA, USA) according to Deckers et al. (2020) in a FLUOstar Omega microplate reader (BMG Labtech, Ortenberg, Germany). Results for each herbicide treatment were expressed in % of the respective untreated plants of the same population to remove daily variability.

#### 2.2.3 Lipid peroxidation

Lipid peroxidation was determined by spectrophotometric detection of malondialdehyde (Hodges et al., 1999). Samples (0.05 g) were homogenised and MDA-TBA complex was extracted as described in Eceiza et al. (2022). Malondialdehyde-TBA complex content was measured at 532 nm and correction for unspecific turbidity and sugar-TBA complexes was done by subtracting the absorbance of the same sample at 600 and 400 nm respectively (Hodges et al., 1999; Verma and Dubey, 2003).

#### 2.2.4 Glutathione and related compounds

Glutathione was extracted from samples (0.1 g) in 1 mL 1 M HCl, derivatised with 5-iodoacetamide fluorescein (Eceiza et al., 2022) and reduced with tributylphosphine (Zinellu et al., 2005). Glutathione content was measured by capillary electrophoresis (CE) equipped with a laser-induced fluorescence detector (Zulet et al., 2015).

Reduced glutathione (GSH) was determined directly by injection of an aliquot in the CE. Oxidised glutathione (GSSG) was reduced with 10% tributylphosphine and then total GSH, cysteine (Cys) and γ-glutamyl-cysteine (GGC) were analysed by CE. GSSG was determined as the difference between both total and reduced GSH values (Eceiza et al., 2022).

#### 2.2.5 Ascorbate

Ascorbate was extracted from samples (0.05 g) in 0.3 mL ice cold 2% meta-phosphoric acid containing 1 mM EDTA (Schützendübel et al., 2002). The homogenate was filtered (0.22 μm, Millex GV),. Ascorbate was analysed by high-performance CE in a Beckman Coulter P/ACE MDQ (Fullerton, CA, USA) associated with a diode array detector (Herrero-Martínez et al., 2000) and equipped with the P/ACE station software for instrument control and data handling (Eceiza et al., 2022).

Reduced ascorbate was determined directly by injecting an aliquot in the CE. Dehydroascorbic acid was reduced with 200 mM dithiothreitol and then total ascorbate was analysed by CE. Dehydroascorbic acid was determined as the difference between both ascorbate values (Eceiza et al., 2022).

#### 2.2.6 Enzymatic activities

For the enzymatic activities, proteins were extracted from samples (0.1 g) as described in Eceiza et al. (2022). Activities of four antioxidant enzymes were measured according to Eceiza et al. (2022): superoxide dismutase (SOD), catalase (CAT) peroxidase (POX) and glutathione reductase (GR). SOD activity was measured as the disappearance of superoxide formed by the reaction between xanthine and xanthine oxidase. Increase in absorbance due to the disappearance of superoxide was measured at 550 nm. CAT activity was measured as the disappearance of H_2_O_2_. Decrease in absorbance owing to the disappearance of H_2_O_2_ was measured at 280 nm. Activity of peroxidases was measured with guaiacol as the substrate. Increase in the absorbance due to oxidation of guaiacol was measured at 470 nm. GR activity was measured with NADPH as the substrate. Decrease in absorbance due to the oxidation of NADPH was measured at 340 nm. Results of the four enzymatic activities were related to total soluble protein, measured in the same extracts (Bradford, 1976). A synergy™ HT Multi-Detection Microplate Reader (BioTek Instruments Inc., Winooski, VT, USA) was used for absorbance measurements.

#### 2.2.7 Gene expression analysis

Relative transcript level was calculated for three genes encoding antioxidant enzymes, corresponding to two isozymes of SOD (*CuZnSOD* and *MnSOD*) and CAT (*CAT*).

RNA extraction and the subsequent cDNA synthesis were performed according to Fernández-Escalada et al. (2017). The cDNA was diluted tenfold and stored at -20 °C until analysis as described in Hendrix et al. (2020). Primers (**Supplementary Table 1**) were designed using the coding sequence of *A. palmeri* and crossed with *Amaranthus hypochondriacus* in the case of both SOD isozymes and *Spinacia oleracea* in the case of *CAT*, using Primer 3 software. Their specificity was verified in silico using NCBI BLAST.

Quantitative real-time PCR (qPCR) was performed using the Quantinova™ SYBR^®^ Green PCR Kit (Qiagen, Hilden, Germany) and according to Hendrix et al. (2020). The amplification conditions were the following: 2 min at 95 °C, 60 cycles of 5 s at 95 °C and 25 s at the annealing temperature (**Supplementary Table 1**). In order to confirm product specificity, a melt curve was generated. Relative gene expression levels were calculated using the 2^-ΔCq^ method. Data were normalised against the expression of a set of stable reference genes selected *via* the GrayNorm algorithm (Remans et al., 2014) listed in **Supplementary Table 1**; and whose primers were designed using the sequence of *A. palmeri* and crossed with *A. hypochondriacus*. Primer efficiencies were obtained using a standard curve of a two-fold dilution series generated from a pooled sample. The optimal annealing temperature (58-60 °C) for each primer was determined by gradient PCR, and only primers with an efficiency between 80 and 120% were used for qPCR analysis.

### 2.3 Statistical analysis

Separately maintained individual plants were considered as biological replicates. For statistical analyses, plants were separated according to their resistance level: sensitive (no mutations in *ALS*) and resistant (mutations in Trp-574 or Trp-574+Ser-653). Each mean value was calculated using samples from 4 different individual plants with the same genotype (sensitive or resistant) that received the same herbicide treatment. Possible difference between untreated (control) plants of each population was evaluated using Student’s t-test and considered significant when p-value ≤ 0.05. In each population, the differences between plants that had received a herbicide treatment (FR of nicosulfuron or 3FR of nicosulfuron) and the untreated plants of the same population were evaluated using Student’s t-test and considered significant when p-value ≤ 0.05. Significant differences between untreated plants of both populations are highlighted in the figures with pound symbols. Significant differences between untreated and treated plants within each population are highlighted in the figures with asterisks.

All statistical analyses were carried out using SPSS 27 software. Graphs were constructed using Sigma Plot 12 software.

## 3 Results

Untreated S and R plants grew equally. Treated S plants were alive one week after treatment (when leaves were sampled), but died in the following few weeks, as a result of a slow herbicidal response. Resistant plants did not die in response to herbicide treatment.

### 3.1 Genotyping of R plants (dCAPS)

The resistance genotyping of the resistant individuals is shown in **Table 1**. All R plants contained the Trp-574 mutation and were heterozygous for this trait, so it can be established as the main mutation conferring resistance in this population. An example of a representative agarose gel showing the detection of heterozygosity by dCAPS is shown (**Supplementary Figure 1**).

**Table 1:** Genotyping of resistant (R) *Amaranthus palmeri* plants original from Lleida (Catalonia, Spain).

Mutation type	Mutation	% of total R (mutated) individuals	Heterozygous individuals (Trp-574) (%)	Homozygous individuals (Trp-574) (%)	Heterozygous individuals (Ser-653) (%)	Homozygous individuals (Ser-653) (%)
Simple	Trp-574	95.24	95	5	–	–
Simple	Ser-653	0	–	–	–	–
Double	Trp-574 + Ser-653	4.76	100	0	50	50

Percentages of plants with each mutation(s) with respect to total resistant plants; and of heterozygous individuals for each mutation with respect to all plants containing that mutation are shown.

Most plants showed the Trp-574 mutation as a single mutation, but some plants also contained the Ser-653 mutation, being then resistant due to a double mutation. While the genotyping of the resistant population was based on almost 100 individuals, the individuals used for the physiological study were selected to have only the Trp-574 mutation, ensuring the genetic similarity of the population.

### 3.2 H_2_O_2_ and MDA content

**Figure 1:**
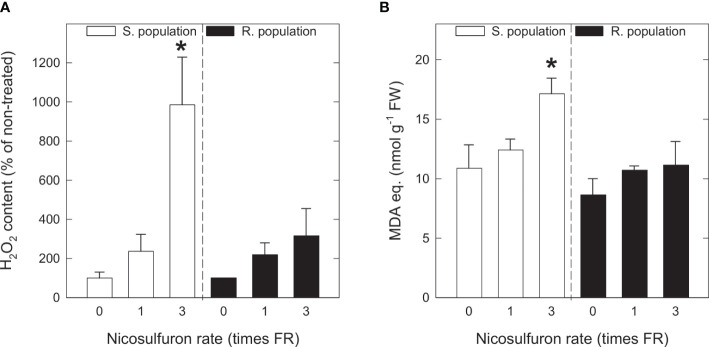
H_2_O_2_ content **(A)** and malondialdehyde (MDA) equivalents **(B)** in *Amaranthus palmeri* sensitive (S, white) and resistant (R, black) populations treated with different nicosulfuron rates (X axis, times recommended field rate or FR, FR = 0.06 kg ha^-1^). Mean ± SE (n = 4). For each population, significant differences between treatments and their respective untreated are highlighted with asterisks (Student’s t-test, p value ≤ 0.05).

There was a similar H_2_O_2_ concentration in untreated S and R plants (**Supplementary Table 2**). Nicosulfuron treatment induced H_2_O_2_ accumulation, especially in S plants treated with 3FR (**Figure 1A**). For its part, MDA basal levels (of untreated plants) were similar in S and R plants (**Figure 1B**). MDA content increased proportionally to nicosulfuron rate in S plants, with a significant MDA accumulation in plants treated with 3FR (**Figure 1B**). In R plants, however, no significant changes were detected.

### 3.3 Non-enzymatic antioxidants

**Figure 2:**
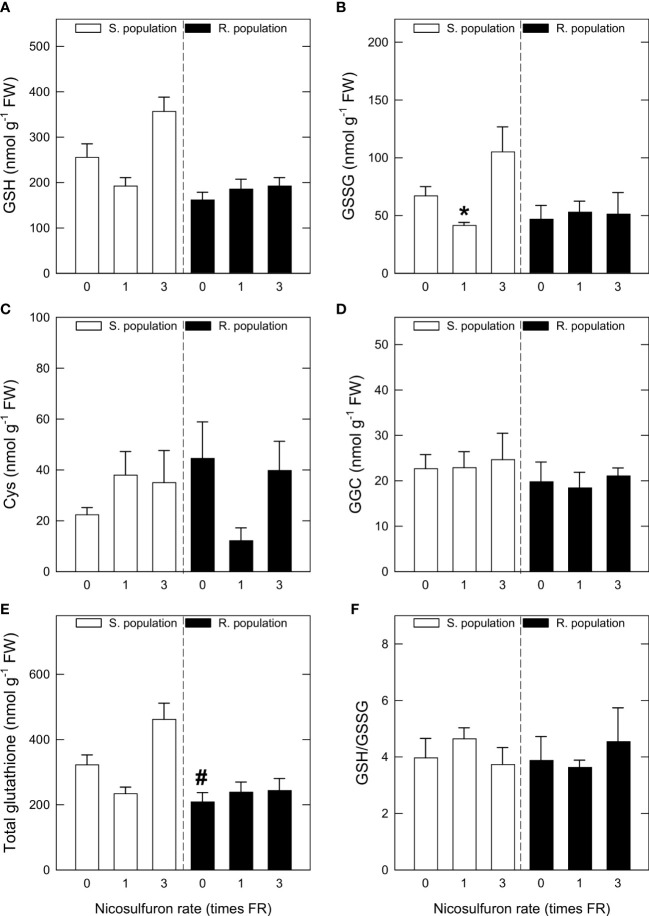
Reduced glutathione (GSH) content **(A)**, oxidised glutathione (GSSG) content **(B)**, γ-glutamyl-cysteine (GGC) content **(C)**, Cys content **(D)**, sum of GSH and GSSG (total glutathione) contents **(E)** and GSH to GSSG ratio **(F)** in *Amaranthus palmeri* sensitive (S, white) and resistant (R, black) populations treated with different nicosulfuron rates (X axis, times recommended field rate or FR, FR = 0.06 kg ha^-1^). Mean ± SE (n = 4). Significant differences between untreated S and R plants are highlighted with pound symbols (Student’s t-test, p value ≤ 0.05). For each population, significant differences between treatments and their respective untreated are highlighted with asterisks; (Student’s t-test, p value ≤ 0.05). "#" indicates pound.

Untreated S plants showed a higher total GSH content than R plants, more due to the GSH content than to the GSSG content (**Figures 2A, B, E**). Both GSH and especially GSSG contents dropped with FR in S plants, a tendency that may also be seen in Cys content in R plants (**Figure 2C**), although in both cases no significant differences were found. GGC content and GSH to GSSG ratio did not show any significant variations between untreated and treated plants (**Figures 2D, F**) and none of these parameters changed in response to nicosulfuron in R plants.

**Figure 3:**
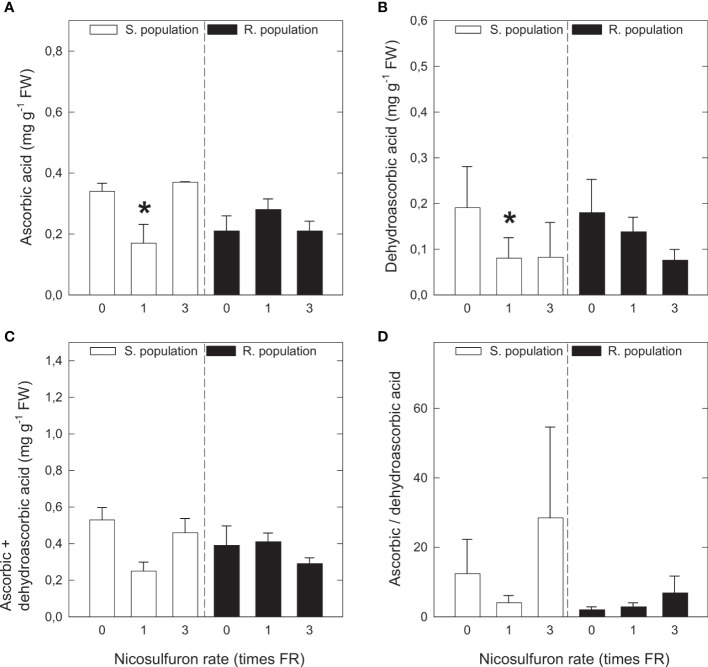
Ascorbic acid content **(A)**, dehydroascorbic acid content **(B)**, sum of ascorbic acid and dehydroascorbic acid **(C)** and ascorbic acid to dehydroascorbic acid ratio **(D)** in *Amaranthus palmeri* sensitive (S, white) and resistant (R, black) populations treated with different nicosulfuron rates (X axis, times recommended field rate or FR, FR = 0.06 kg ha^-1^). Mean ± SE (n = 4). For each population, significant differences between treatments and their respective untreated are highlighted with asterisks (Student’s t test, p value ≤ 0.05).

Nicosulfuron treatment did not induce remarkable changes in ascorbic acid and dehydroascorbic acid content (**Figure 3**). Ascorbic acid content showed a decreasing tendency in S plants with FR, but with 3FR it was very similar to that of the control (**Figure 3A**). The pattern was the same for dehydroascorbic acid (**Figure 3B**) the sum of ascorbic acid and dehydroascorbic acid (**Figure 3C**) and ascorbic acid to dehydroascorbic acid ratio (**Figure 3D**). None of the analysed parameters showed any variation in R plants.

### 3.4 Antioxidant enzymatic activities and gene expressions

**Figure 4:**
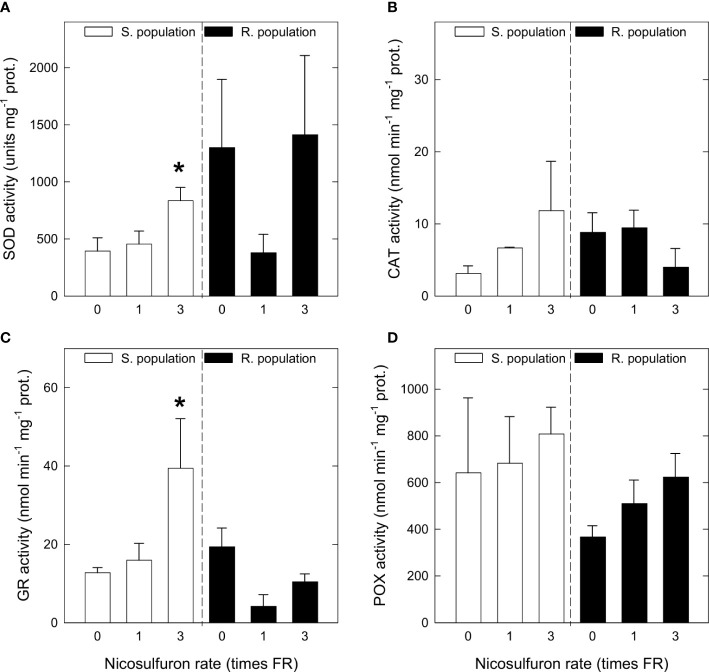
Superoxide dismutase (SOD) activity **(A)**, catalase (CAT) activity **(B)**, glutathione reductase (GR) activity **(C)** and peroxidases (POX) activity **(D)** in *Amaranthus palmeri* sensitive (S, white) and resistant (R, black) populations treated with different nicosulfuron rates (X axis, times recommended field rate or FR, FR = 0.06 kg ha^-1^). Mean ± SE (n = 4). For each population, significant differences between treatments and their respective untreated are highlighted with asterisks (Student’s t-test, p value ≤ 0.05).

Activities of four antioxidant enzymes were measured: SOD, CAT, GR, and POX. SOD and CAT are important antioxidant enzymes that scavenge superoxide and H_2_O_2_, respectively. GR is a key enzyme in the glutathione-ascorbate cycle that catalyses GSH turnover *via* GSSG reduction (Carlberg and Mannervik, 1985), while peroxidases conform a vast group of enzymes with many different functions, including antioxidant activity (Radwan and Fayez, 2016). Basal enzymatic activities of S and R plants were statistically similar (**Figure 4**). SOD (**Figure 4A**) and GR (**Figure 4C**) activities increased in S plants with herbicide treatment, being the increases significant with 3FR; in R plants no significant changes were observed. CAT showed a similar increasing tendency in S plants, and a decreasing tendency in R plants, but in none of these cases was it significant (**Figure 4B**). POX tended to increase proportionally to nicosulfuron rate in both populations, yet it was not significant (**Figure 4D**).

**Figure 5:**
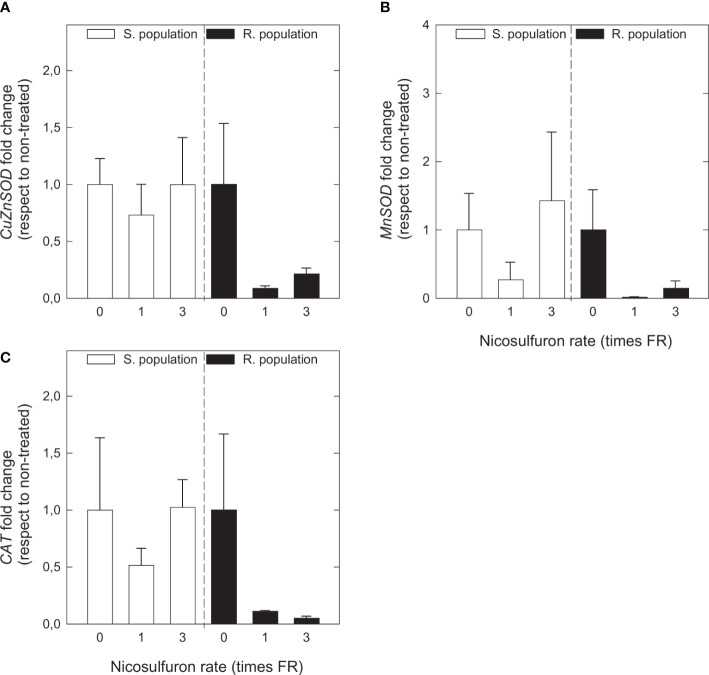
CuZn superoxide dismutase (*CuZnSOD*) gene expression **(A)**, Mn superoxide dismutase (*MnSOD*) gene expression **(B)** and catalase (*CAT*) gene expression **(C)** in *Amaranthus palmeri* sensitive (S, white) and resistant (R, black) populations), in relative fold change with respect to untreated plants of each population, treated with different nicosulfuron rates (X axis, times recommended field rate or FR, FR = 0.06 kg ha^-1^). Mean ± SE (n = 4). For each population, no significant differences between treatments and their respective untreated were observed (Student’s t test, p value ≤ 0.05).

Regarding gene expressions, relative transcript levels were calculated for three genes encoding two isozymes of superoxide dismutase (*CuZnSOD* and *MnSOD*) and catalase (*CAT*). Both SOD and CAT are important antioxidant enzymes that act as superoxide and H_2_O_2_ (two of the main ROS) scavengers, respectively. Superoxide dismutase isozymes differ in the location: CuZnSOD is found in cytosol, chloroplasts and peroxisomes and MnSOD is found in mitochondria and peroxisomes (Houmani et al., 2016). Firstly, untreated plants of both populations were compared and then, for treated plants in each population, the relative copy number (fold change) with respect to the respective untreated samples was calculated. None of the three analysed genes (*CuZnSOD*, *MnSOD*, and *CAT*) presented remarkable differences between untreated S and R plants (**Supplementary Table 2**). Gene expressions of the three of them were similar in the S population independently of nicosulfuron rate, and they tended to decrease with nicosulfuron rate in the R population, but no significant differences were observed (**Figure 5**).

## 4 Discussion

### 4.1 Resistance confirmation: Trp-574, the main mutation

According to Torra et al. (2020), resistance of resistant plants was conferred by Ser-653 and Trp-574 mutations. In this study, the Trp-574 mutation was found in all resistant plants and, in most of them, it was present as a heterozygous single mutation. In addition, in the 5% of the resistant individuals, Trp-574 mutation was complemented with the Ser-653 mutation. In order to minimise the genetic variability of the resistant plants, the physiological study was performed with plants only containing the Trp-574 mutation.

Trp-574 substitution is the most common point mutation that confers resistance to ALS inhibitors (Molin et al., 2016; Singh et al., 2019; Torra et al., 2020) and it has been found in populations of many other weeds including grasses (Yu et al., 2010; Hamouzová et al., 2014; Hernández et al., 2015; Deng et al., 2017; Mei et al., 2017; Wang et al., 2019). It is known to confer a broad cross-resistance to several chemical families of ALS inhibitors apart from sulfonlyureas, such as imidazolinones and triazolpirimidines (Yu et al., 2012; Küpper et al., 2017).

The mutant allele is dominant, and is not supposed to alter ALS functionality significantly (Yu et al., 2010). Moreover, according to previous studies, Trp-574 mutation does not endow the plants with additional physiological traits, as mutated and unmutated plants show similar growth and total amino acid, soluble sugar, and starch contents (Barco-Antoñanzas et al., 2022). This means that the mutation confers an important cross-resistance to ALS inhibitors with apparently negligible fitness costs (Tranel and Wright, 2002). Thus, as long as selection pressure made by ALS-inhibiting herbicides in fields continues, the prevalence of the Trp-574 mutation and hence the proportion of ALS inhibitor-resistant individuals is expected to rise, making resistance to ALS inhibitors an increasingly serious issue.

The present study showed that Trp-574 mutation does not have implications in the basal oxidative state either, as untreated S and R plants showed similar values for almost all the analysed parameters. Similar basal MDA and antioxidant levels in S and R plants mean that herbicide resistance is not accompanied by a different oxidative profile or an intrinsically enhanced antioxidant system, and there are no changes associated to the oxidative status in the R plants with respect to the S ones. The resistance is restricted to just the herbicide target (ALS enzyme), so any side effect of nicosulfuron that is not linked to ALS inhibition should occur in both sensitive and resistant plants.

### 4.2 ALS inhibition causes oxidative damage in sensitive plants treated with high herbicide rates

That nicosulfuron has the potential of causing oxidative stress is made clear by the H_2_O_2_ accumulation induced by the herbicide treatment, which is especially remarkable in the S plants treated with 3FR. Hydrogen peroxide is an ubiquitous ROS synthesised in many physiological processes with plenty of biological functions, however, its formation usually increases under stress conditions and induces oxidative damage, mainly through hydroxyl (•OH) formation (Demidchik, 2015). This H_2_O_2_ accumulation following nicosulfuron treatment has been observed before in other species, especially in maize (Wang et al., 2018a; Wang et al., 2018c; Liu et al., 2019; Yang et al., 2021). In *A. palmeri*, a significant accumulation only occurred in S plants. The fact that resistance is strictly based on the ALS enzyme and is not accompanied by a different oxidative metabolism, evidences that H_2_O_2_ formation is mainly due to ALS inhibition.

Physiological effects elicited by ALS inhibition have been related to oxidative stress beforehand, but the exact mechanisms induced by ALS inhibition that end ROS production are still unknown. One hypothesis is the activation of hypoxic metabolism (fermentation) in response to herbicide treatment, owing to a lower carbohydrate usage in roots (Gaston et al., 2002; Zabalza et al., 2005). In hypoxic tissues, H_2_O_2_ formation occurs because of over-reduction of redox chains (Blokhina et al., 2003). Another possible explanation for ROS formation after ALS inhibition would be residual respiration. Plants treated with ALS inhibitors are supposed to display higher residual respiration, increasing oxygen consumption and giving rise to a greater proportion of oxygen available to be used in the formation of ROS (Zabalza et al., 2007).

MDA is the main indicator of lipid peroxidation, and its accumulation is an almost unequivocal sign of oxidative damage. MDA accumulation in response to nicosulfuron treatment has been reported before in various species (Wang et al., 2018a; Liu et al., 2019; Silva et al., 2020; Concato et al., 2022); and it has been reported in pea leaves treated with imazethapyr, another ALS-inhibiting herbicide (Zabalza et al., 2007). In the present study, MDA increased proportionally to nicosulfuron rate in S plants, with a significant accumulation in plants treated with 3FR, and no major changes in R plants. These results are very consistent with the observed H_2_O_2_ accumulation and confirm a moderate oxidative damage in sensitive plants treated with 3FR.

### 4.3 Disparate activation of antioxidant systems in response to herbicide treatment

Moving into antioxidant systems, there was not a clear pattern in GSH contents and precursors, and in neither of the two populations is glutathione synthesis significantly enhanced by herbicide treatment, although there is an increasing tendency in S plants treated with 3FR. Nevertheless, there is some evidence that glutathione is acting as an endogenous antioxidant in treated S plants since GR activity strongly increases in S plants treated with 3FR. Glutathione’s antioxidant activity is based on the reduction of H_2_O_2_ and peroxidised lipids, both of which are highly accumulated in S plants treated with 3FR. In return, GSH is oxidised to GSSG. Glutathione reductase, which follows the same pattern as the oxidative damage markers (H_2_O_2_ and MDA accumulation), catalyses GSSG to GSH reduction (Prego, 1995; Couto et al., 2016). Thus, it is likely that, in S plants treated with 3FR, glutathione’s antioxidant activity is much higher, but no decrease in GSH content in favour of GSSG is observed due to a hugely increased GR activity.

Although ascorbic acid content decreased in S plants with FR, dehydroascorbic acid also decreased with the same rate, so the oxidative status of the plant is not altered in this sense. Furthermore, in plants treated with 3FR, the only rate with which a clear oxidative stress is induced, values for both compounds are similar to the ones in untreated plants. In R plants, no significant changes in antioxidant systems were detected, showing that R plants do not suffer oxidative stress after nicosulfuron application, and hence reinforcing the hypothesis that oxidative stress induced in S plants is related to ALS inhibition.

Activity of peroxidases could have been expected to increase, as a peroxidase (exactly glutathione peroxidase) is the enzyme that catalyses GSH oxidation (Prego, 1995). Nevertheless, only a slight, non-significant increase occurred, possibly because peroxidases are a very diverse group of enzymes involved in many physiological processes apart from the antioxidant system, such as germination, cell elongation, and lignification (Shigeto and Tsutsumi, 2016).

About the other two enzymes, SOD and CAT, what was observed with enzymatic activities was different to the observed in gene expression. Enzymatic activities of both enzymes tended to increase with nicosulfuron rate in S plants, although the increase was only significant in the case of SOD. This enzyme is a major superoxide scavenger. Superoxide is a more reactive and much less stable ROS than H_2_O_2_, but both ROS are strongly related (Demidchik, 2015). In view of the accumulation of H_2_O_2_ and the increase of SOD activity in S plants treated with 3FR, it is likely that superoxide formation is also increased in these plants. For its part, the increase of CAT activity with nicosulfuron rate in S plants was less clear, and in R plants it even showed a decreasing tendency. The cause of non-significance in S plants and the decreasing tendency in R plants could be a slight enzymatic inhibition caused by herbicidal activity. Concato et al. (2022) investigated the effects of various ALS inhibitors, including nicosulfuron, on the antioxidant system of *Brassica napus* and found that CAT was inhibited by all of them, similar to the study by Qian et al. (2011) in *Arabidopsis thaliana*. The difference in behaviour between the two populations is probably due to the huge H_2_O_2_ accumulation in S plants. Regarding gene expressions of *CuZnSOD*, *MnSOD* and *CAT*, both SOD isozymes and CAT behaved similarly, not following a clear pattern in S plants and with a tendency to decrease with nicosulfuron rate in R plants. This evidences that, in response to oxidative stress caused by nicosulfuron, SOD and CAT are induced posttranscriptionally.

These results do not entirely match with results from other species, especially *Zea mays*, where several studies have observed a clear activation of the antioxidant systems in response to nicosulfuron treatment (Wang et al., 2018a; Wang et al., 2018c; Liu et al., 2019; Yang et al., 2021). Biochemical and physiological differences between *Z. mays* and *A. palmeri* might explain the differences in the antioxidative response. Maize has a variable tolerance to sulfonylurea herbicides, which is not based on ALS but on a rapid metabolic inactivation by hydroxylation on the pyrimidine ring followed by glucose conjugation, with an important involvement of cytochrome P450 (Wittenbach et al., 1994; Dragičević et al., 2012). This sulfonylurea-resistance mechanism has been associated with oxidative metabolism, probably involving an important antioxidant induction (Wang et al., 2018b).

### 4.4 Toxicity of ALS inhibitors is independent of oxidative stress

Finally, the most remarkable effect of nicosulfuron was oxidative damage in S plants, caused by ROS overproduction. Given that ROS formation was related to ALS inhibition, it can be taken as a general effect of ALS-inhibiting herbicides. The physiological mechanisms that link ALS inhibition with ROS formation are yet to be demonstrated and make up a potential object of study.

However, oxidative damage was only significant in S plants treated with 3FR (at least one week after treatment), when FR was already lethal for these plants after a couple weeks. This evidences that, as proposed before (Zabalza et al., 2007), oxidative damage is not one of the main causes of plant death. ALS-inhibiting herbicides trigger a moderate oxidative stress through ALS inhibition, but this oxidative stress is independent of herbicide toxicity.

## 5 Conclusion

To sum up, the resistance mechanism to ALS-inhibiting herbicides in the studied *A. palmeri* population is restricted to the target and is not accompanied by a different oxidative status. In response to high (supralethal) rates of nicosulfuron, sensitive plants suffer oxidative damage related to ALS inhibition. This oxidative damage is palliated, somewhat owing to the antioxidant role of glutathione and the antioxidant enzymes, and is not one of the main causes of plant death. Resistant plants do not suffer oxidative damage nor increase their antioxidant activity. Having concluded that triggered oxidative stress is due to ALS inhibition, the presented results are surely extensible to other ALS-inhibiting herbicides to which plants with Trp-574 mutation are resistant. However, other plants species or plants with different resistance mechanisms may respond differently.

## Data availability statement

The raw data supporting the conclusions of this article will be made available by the authors, without undue reservation.

## Author contributions

MR and AZ conceived the study. AZ, AC, and MR designed the experiments. MB-A, ME, and MH performed the experiments and analysed the data. ME wrote the draft of the manuscript. MG-M, MH, AZ, AC, and MR revised the manuscript. ME wrote the final version of the manuscript. All authors read and approved the final manuscript.
