# Supplementary material for: Role of oxidative stress in the physiology of sensitive and resistant Amaranthus palmeri populations treated with herbicides inhibiting acetolactate synthase
Source: Front Plant Sci. 2023 Jan 6;13:1040456. doi: 10.3389/fpls.2022.1040456 (PMC9852854; doi:10.3389/fpls.2022.1040456)
Supplement: Supplementary file 1 [file DataSheet_1.pdf]

Supplementary Material

Suppl. Table 1. Primers used for gene expressions of CuZn superoxide dismutase (*CuZnSOD*), Mn superoxide dismutase (*MnSOD*) and catalase (*CAT*); and reference genes β-tubuline (*β-Tub*), Peter Pan-like protein (*PPAN*), actin (*Actin*), ubiquitin conjugation enzyme (*UCE*), glyceraldehyde-3-phosphate dehydrogenase (*GADPH*) and carbamoyl-phosphate synthetase (*CPS*). Forward and reverse primer sequences and their respective annealing temperatures are shown.

Gene	Primer sequence (forward)	Primer sequence (reverse)	Ann. T (ºC)
CuZnSOD	TCCTTCTCTCTTCTCCAGGG G	TGTCACCAAGGGCATG AACA	60
MnSOD	GGCGAAATCATGCAGCTTC A	TGCATCAATAGCCCACC CAA	60
CAT	TAATGCTCCCAAGTGCCCT C	TCTCTCCCCTGGTTCCT TGA	58
β-Tub	ATGTGGGATGCCAAGAAC ATGATGTG	TCCACTCCACAAAGTAG GAAGAGTTCT	58
PPAN	TGCTCCATTTTTGAGGGTT GC	GACATCGAGGCCTCAA CTGTG	60
Actin	ACTTCGAGTTGCACCTGAG G	AGAATAGCGTGGGGAA GTGC	58
UCE	GGCACTATTCCTGGTCCTG T	AGATCGCCCCACTCTGA CT	60
GADPH	GTTTTGCCAGCTCTCAACG G	CATCATCCTCGGTGTAG CCC	58
CPS	ATTGATGCTGCCGAGGATA G	GATGCCTCCCTTAGGTT GTTC	60

Suppl. Table 2. Absolute values for untreated sensitive (S, left) and resistant (R, right) plants for H_2O_2 content, CuZn superoxide dismutase (*CuZnSOD*) gene expression, Mn superoxide dismutase (*MnSOD*) gene expression and catalase (*CAT*) gene expression. Mean ± SE (n = 4). No significant differences between populations were observed (Student's t test, p value ≤ 0.05).

	Untreated S	Untreated R
H_2O_2 content (nmol g^{-1} FW)	27 ± 10	17.2 ± 6.9
CuZn superoxide dismutase (*CuZnSOD*) gene expression (normalised RQ)	0.94 ± 0.21	1.0 ± 0.5
Mn superoxide dismutase (*MnSOD*) gene expression (normalised RQ)	0.37 ± 0.20	0.43 ± 0.17
Catalase (*CAT*) gene expression (normalised RQ)	1.0 ± 0.6	0.70 ± 0.20

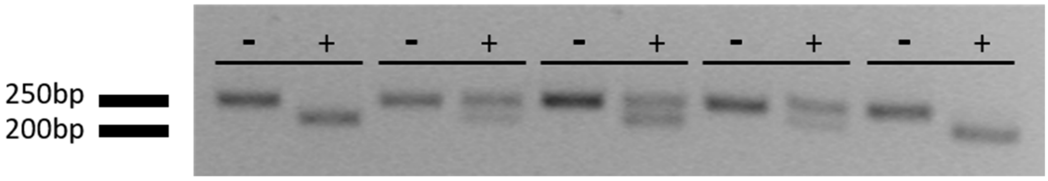

Suppl. Fig. 1. A model agarose gel used for Derived Cleaved Amplified Polymorphic Sequences (dCAPS) assay for the Trp-574 mutation of the ALS gene. For each sample, one replicate was incubated with the restriction enzyme NcoI (+), and another replicate was not incubated with the restriction enzyme and served as reference (-). NcoI-restricted fragments (230bp) and NcoI-unrestricted fragments (256bp) correspond to wild and mutant alleles, respectively. Heterozygous plants contain both restricted and unrestricted fragments. The first and the last samples in the gel belong to sensitive plants and the three in the middle belong to heterozygous resistant plants.
